# Decoding the Complex Crossroad of Tryptophan Metabolic Pathways

**DOI:** 10.3390/ijms23020787

**Published:** 2022-01-12

**Authors:** Giada Mondanelli, Claudia Volpi, Ciriana Orabona

**Affiliations:** Department of Medicine and Surgery, University of Perugia, 06132 Perugia, Italy; claudia.volpi@unipg.it

Among the 20 amino acids needed for protein synthesis, Tryptophan (Trp) is an aromatic amino acid fundamental not only for the synthesis of the major components of living cells (namely, the proteins), but also for the maintenance of cellular homeostasis. During the evolution, most organisms stopped producing 9 of 20 amino acids and thus became auxotroph for these so-called essential amino acids, which include, among others, Trp. As opposed to mammals that are addicted for an external supply, several microorganisms and higher plants are endowed with the machinery to produce Trp from chorismate, a common precursor of aromatic amino acids produced by the shikimate pathway from phosphoenol pyruvate and erythrose-4-phosphate [1,2]. Although sharing the common substrate, mammals, microbes and plants utilize different metabolic pathways to generate a plethora of Trp-derived metabolites. In mammals, the majority of Trp is degraded along the kynurenine pathway, from the name of the first generated metabolite (i.e., l-kynurenine; l-kyn), through a rate-limiting step catalyzed by the enzymes indoleamine 2,3-dioxygenase (IDO1 and IDO2) and tryptophan 2,3-dioxygenase (TDO2). Only a small proportion of Trp is metabolized into serotonin via two consecutive reactions catalyzed by the enzymes tryptophan hydroxylase and 5-hydroxytryptophan decarboxylase [3]. The commensal microbes of the skin, pulmonary, genitourinary and gastrointestinal tracts can enzymatically transform Trp into indole-derivatives (including indole 3-acetic acid, indole-3-aldehyde and indole-3-carboxaldehyde) and tryptamine, while the higher plants can degrade Trp mainly into indoleamines (such as serotonin and melatonin) and auxin (namely, indole containing molecules). The Trp-derived metabolites affect several physiologic processes ranging from immunity, nervous system and gastrointestinal functions as well as every stage of the plant life cycle, including germination, seedling development and vegetative growth [2,4,5]. Mammals and microbes have become particularly apt to exploit the Trp metabolism to control each other. Indeed, mammals, by increasing the amino acid catabolism, can restrict the local Trp availability to invading pathogens and thus control the infection. Noteworthy, this strategy has also become a means of fine-tune the host’s own reactivity in response to infection and self-antigens, and thus to avoid hyperinflammation and autoimmunity. On their side, some microbes as well as transformed tumor cells can exploit the host’s machinery of Trp degradation to escape the immune control and thus infect or grow undisturbed, respectively.

Given its pleiotropic functions, an altered Trp metabolism is associated with a myriad of pathologic conditions and the enzymes, the metabolites themselves, or their receptors have thus emerged as promising therapeutic targets. A variety of Trp-derived metabolites have been demonstrated to activate the Aryl hydrocarbon Receptor (AhR) for transducing an immunosuppressive signal in the tumor microenvironment [6]. As able to relieve the immune suppression, small molecule inhibitors targeting the IDO1 and TDO2—usually over activated in cancer cells—are under preclinical and clinical evaluation for the treatment of neoplasia [7]. Many kynurenines exert neuroprotective or neurotoxic effects in part through their effects on N-methyl-D-aspartate receptor (NMDA) signaling and glutamatergic neurotransmission. Alteration of kynurenine pathway is indeed associated with psychiatric illness, depression, schizophrenia-like cognitive deficits and pain [8]. As a matter of the fact, an impaired activity of glutamatergic transmission as well as an increased levels of brain kynurenic acid (KYNA) have been observed in schizophrenia. Conventional inhibitors of the active site of kynurenine aminotransferase (KAT)—the enzyme producing KYNA from l-kyn—and irreversible inhibitors of the co-factor pyridoxal 5′-phophate (PLP) have been developed. However, given the potential toxicity and lack of significant efficacy, these molecules did not reach the clinical trials. A central function for PLP (which is the active form of vitamin B6) across Trp metabolism have been proposed to modulate the host-microbiota crosstalk [9]. Indeed, in both mammals and microbes, several enzymes catalyzing the critical steps of Trp catabolism and synthesis use PLP as co-factor, which thus emerges as a common denominator that may direct the generation of Trp metabolite over the one other. As dysregulated Trp metabolism may unbalance the production of metabolites with potential pathological consequences, the ability to manipulate the Trp flux—either towards the host or the microbes—could be therapeutically exploited to restore homeostatic conditions. Therefore, it’s conceivable that vitamin B6 may reach the landscape of agents acting on Trp metabolism, as by affecting the PLP availability, it can turn on/off either microbial or host’s enzymes and thus direct the production of specific metabolites [9].

As the glutamate signaling through the NMDA receptor in the spinal sensory neurons is involved in the generation of hypersensitivity and pain sensation, Trp-metabolites acting as NMDA agonist (QA, quinolinic acid) or antagonist (KYNA) can regulate neuropathic pain and depressive behavior, besides epilepsy and neurodegeneration. Pharmacologic inhibition of kynurenine 3-monooxygenase (KMO; the enzyme responsible for the generation of 3-hydroxykynurenine, 3-HK) shifts the Trp-metabolism toward the production of neuroprotective KYNA, and thus alleviates both neuropathic pain and neuropathic pain-induced depression [10]. The inhibition of not only KMO, but also of IDO1, IDO2 and Kynureninase (being over activated in the tissues of central nervous system of different neuropathic pain models) results in a decreased mechanical, tactile, and thermal hypersensitivity. Although these observations are still limited to preclinical models, they open a new perspective for the treatment of chronic pain conditions by targeting specific enzymes of the kynurenine pathway and the metabolites thereof. A shift of the local and systemic Trp metabolism from the neuroprotective KYNA toward the neurotoxic 3-HA has been associated with the neurodegeneration of both retinal ganglion cell and other vision-related brain structures that leads to glaucomatous manifestation (i.e., a progressive optic neuropathy that can cause irreversible blindness) [11]. Thus, blocking the generation of 3-HA by means of specific KMO inhibitors could be therapeutically advantageous even for the treatment of glaucoma. However, as opposed to IDO1 inhibitors that have already reached clinical trials, KMO inhibitors—albeit promising drugs in neurodegenerative diseases—are still in the earlier preclinical stage of development.

Alternatively to enzyme inhibitors (that have largely gained space in the treatment of cancers, metabolic and neurological diseases), supplementation of Trp or specific metabolites as well as activation of enzymes of the kynurenine pathway could be beneficial for relieving pathologies associated with defective Trp metabolism [12]. Being well-tolerated, dietary Trp supplementation could help in fighting symptoms of inflammatory bowel diseases, in increasing sleep efficiency and reducing anxiety and depression symptoms, and in slowing down the progression of chronic kidney disease (CDK). A gut-brain axis in which Trp serves as metabolic substrate of gut microbiota and the derived metabolites modulate the neural activity, has been well documented [13]. Trp supplementation can also prevent hypertension induced by maternal CKD in adult offspring, through the regulation of gut microbiota compositions and the activation of AhR-mediated signaling pathways [14]. By restoring the *Lactobacillus* abundance, Trp directs its own gut metabolism into indole-aldehyde that, in turn, activates AhR and thus favors the lowering of blood pressure. This opens the scenario to past and next generation of probiotics (live microorganisms, such as Lactobacilli, producing beneficial metabolites), prebiotics (substrates that are selectively utilized by host microorganisms) and postbiotics (non-viable bacteria products or metabolic by-products, such as indole-3-carboxaldehyde), all of which modulate the microbial signatures, especially via Trp metabolism, and confer an health benefit on the host. In this scenario, the production of Kyn in plants of *Nicotiana tabacum* transfected with human IDO1 appears extremely exciting [15].

The special issue “Decoding the Complex Crossroad of Tryptophan Metabolic Pathways” highlights the overarching nature of Trp metabolism, by bringing together the neuropathic pain with the glaucomatous neurodegeneration; the well-known, yet to be still deeply explored, host and microbial interactions with the ability of Trp and its metabolites to prevent hypertension; and the widely explored immune-escaping of tumors in which the serum Kyn/Trp ratio can work as a biomarker for a personalized therapy [16]. From this special issue, it appears clear that Trp metabolism is something far beyond the well-characterized mechanism of immune regulation, which is associated with the auxotrophy of immune cells for the amino acid and with the generation of non-inert metabolites, in both mammals and microbes. Thus, is not surprising that Trp metabolism, which includes Trp itself, metabolites thereof, enzymes and receptors, has emerged as both promising therapeutic target in a wide variety of pathologies and biomarker of disease development and progression. Further investigations into the regulatory mechanisms and interconnections of Trp metabolism within the complexity of human biology will direct the appropriate interventions to restore or maintain homeostatic conditions.
